# Fronto-striatal alterations correlate with apathy severity in behavioral variant frontotemporal dementia

**DOI:** 10.1007/s11682-023-00812-3

**Published:** 2023-10-19

**Authors:** Neeraj Upadhyay, Annika Spottke, Anja Schneider, Daniel C. Hoffmann, Ingo Frommann, Tommaso Ballarini, Klaus Fliessbach, Benjamin Bender, Hauke R. Heekeren, John Dylan Haynes, Michael Ewers, Emrah Düzel, Wenzel Glanz, Laura Dobisch, Katharina Buerger, Daniel Janowitz, Johannes Levin, Adrian Danek, Stefan Teipel, Ingo Kilimann, Matthis Synofzik, Carlo Wilke, Oliver Peters, Lukas Preis, Josef Priller, Eike Jakob Spruth, Frank Jessen, Henning Boecker

**Affiliations:** 1 Deutsches Zentrum für Neurodegenerative Erkrankungen (DZNE), Bonn, Germany; 2 Clinical Functional Imaging Lab, Department of Diagnostic and Interventional Radiology, University Hospital Bonn, Bonn, Germany; 3 Department of Neurology, University Hospital Bonn, Bonn, Germany; 4 Department of Neurodegenerative Disease and Geriatric Psychiatry/Psychiatry, University Hospital Bonn, Bonn, Germany; 5 Department of Diagnostic and Interventional Neuroradiology, University of Tuebingen, Tuebingen, Germany; 6 Department of Education and Psychology, Freie Universität Berlin, Berlin, Germany; 7 Center for Cognitive Neuroscience Berlin, Freie Universität Berlin, Berlin, Germany; 8 Bernstein Center for Computational Neuroscience, Charité-Universitätsmedizin Berlin, Berlin, Germany; 9 Deutsches Zentrum für Neurodegenerative Erkrankungen (DZNE), Munich, Germany; 10 Institute for Stroke and Dementia Research (ISD), University Hospital, LMU Munich, Munich, Germany; 11 Deutsches Zentrum für Neurodegenerative Erkrankungen (DZNE), Magdeburg, Germany; 12 Institute of Cognitive Neurology and Dementia Research (IKND), Otto-von-Guericke University, Magdeburg, Germany; 13 Department of Neurology, University Hospital of Munich, Ludwig-Maximilians-Universität (LMU) Munich, Munich, Germany; 14 Munich Cluster for Systems Neurology (SyNergy), Munich, Germany; 15 Deutsches Zentrum für Neurodegenerative Erkrankungen (DZNE), Rostock, Germany; 16 Department of Psychosomatic Medicine, Rostock University Medical Center, Rostock, Germany; 17 Deutsches Zentrum für Neurodegenerative Erkrankungen (DZNE), Tübingen, Germany; 18 Division Translational Genomics of Neurodegenerative Diseases, Center for Neurology and Hertie-Institute for Clinical Brain Research, University of Tübingen, Tübingen, Germany; 19 Deutsches Zentrum für Neurodegenerative Erkrankungen (DZNE), Berlin, Germany; 20 Department of Psychiatry, Charité-Universitätsmedizin Berlin, Campus Benjamin Franklin, Berlin, Germany; 21 Department of Psychiatry and Psychotherapy, Charité, Berlin, Germany; 22 Department of Psychiatry and Psychotherapy, Klinikum rechts der Isar, Technical University Munich, Munich, Germany; 23 Department of Psychiatry, Medical Faculty, University of Cologne, Cologne, Germany; 24 Excellence Cluster on Cellular Stress Responses in Aging-Associated Diseases (CECAD), University of Cologne, Cologne, Germany

**Keywords:** Behavioral variant FTD, Cortical thickness, Resting state functional connectivity, Neuropsychiatric inventory questionnaire for apathy and disinhibition, Executive dysfunction

## Abstract

Structural and functional changes in cortical and subcortical regions have been reported in behavioral variant frontotemporal dementia (bvFTD), however, a multimodal approach may provide deeper insights into the neural correlates of neuropsychiatric symptoms. In this multicenter study, we measured cortical thickness (CTh) and subcortical volumes to identify structural abnormalities in 37 bvFTD patients, and 37 age- and sex-matched healthy controls. For seed regions with significant structural changes, whole-brain functional connectivity (FC) was examined in a sub-cohort of N = 22 bvFTD and N = 22 matched control subjects to detect complementary alterations in brain network organization. To explore the functional significance of the observed structural and functional deviations, correlations with clinical and neuropsychological outcomes were tested where available. Significantly decreased CTh was observed in the bvFTD group in caudal middle frontal gyrus, left pars opercularis, bilateral superior frontal and bilateral middle temporal gyrus along with subcortical volume reductions in bilateral basal ganglia, thalamus, hippocampus, and amygdala. Resting-state functional magnetic resonance imaging showed decreased FC in bvFTD between: dorsal striatum and left caudal middle frontal gyrus; putamen and fronto-parietal regions; pallidum and cerebellum. Conversely, bvFTD showed increased FC between: left middle temporal gyrus and paracingulate gyrus; caudate nucleus and insula; amygdala and parahippocampal gyrus. Additionally, cortical thickness in caudal, lateral and superior frontal regions as well as caudate nucleus volume correlated negatively with apathy severity scores of the Neuropsychiatry Inventory Questionnaire. In conclusion, multimodal structural and functional imaging indicates that fronto-striatal regions have a considerable influence on the severity of apathy in bvFTD.

**Supplementary Information:**

The online version contains supplementary material available at 10.1007/s11682-023-00812-3.

## Introduction

Frontotemporal dementia (FTD) is the second most common form of dementia in early age (< 65) (Onyike & Diehl-Schmid, 2013), clinically characterized by behavioral impairment, executive dysfunction and language deterioration. Phenotypes with predominant behavioral impairments and primary executive dysfunction are classified as behavioral variant FTD (bvFTD), while patients with language impairment are classified as primary progressive aphasia (Neary et al., 1998). 50–70% of the FTD patients are diagnosed as bvFTD (Johnson et al., 2005).

Studies have assessed the neuroanatomical underpinnings of apathy and disinhibition in bvFTD (Lansdall et al., 2017; Kumfor et al., 2018; Wei et al., 2020; Sheelakumari et al., 2020). Widespread volumetric changes were reported in frontal, temporal, and limbic areas along with cortical thickness (CTh) reductions in frontal, temporal and insular regions (Möller et al., 2016). Functional changes were studied using resting-state functional magnetic resonance imaging (rsfMRI), providing measures of spontaneous brain activity and functional connectivity (FC) (Azeez & Biswal, 2017). Decreased FC in bvFTD was reported within/between lateral prefrontal, basal ganglia, insular, hippocampal and amygdalar regions as well as salience and default mode networks (Ferreira et al., 2022; Kamalian et al., 2022). Conversely, increased FC was reported within the default mode network (Filippi et al., 2013; Whitwell et al., 2011). A relationship between altered mind-wandering capacity and structural / functional integrity of default and frontoparietal networks was reported in bvFTD using a multimodal approach (O’Callaghan et al., 2019). Another multimodal study reported that cortical thinning in temporal and orbitofrontal regions could predict clinical diagnosis of bvFTD (Canu et al., 2017). However, no study has as yet assessed structural alterations to determine seeds regions for analysis of FC and their relationship with neuropsychiatric symptoms within the same bvFTD cohort. Therefore, this study implemented a multimodal approach that first assessed surface-based CTh and subcortical volume changes and, second, examined “seed-to-whole brain” FC from brain areas showing structural alterations in bvFTD patients compared to matched HCs. We hypothesized structural changes in dorsolateral, middle frontal and temporal regions and associated reduced FC linked with behavioral symptoms in bvFTD.

## Methods

Seventy-nine FTD patients were recruited in different clinics affiliated to the multicenter DESCRIBE (DZNE Clinical Register Study of Neurodegenerative Disorders) study at the German Center of Neurodegenerative Diseases (DZNE e.V.). Thirty-seven were diagnosed as bvFTD (Rascovsky et al., 2011) (age (mean ± SD) = 63.81 ± 11.32; sex (M:F) = 21:16) and included in the study. Five patients showed C9orf72 mutations, 1 had VCP and 1 FUS mutations. Three individuals had no DNA samples and the remaining 27 patients did not exhibit any mutations. Diagnostic criteria for bvFTD were presence of any three of clinically characterizing features like apathy/inertia, loss of sympathy, perseverative/compulsive behaviors, hyperorality, disinhibition and executive dysfunction (Rascovsky et al., 2011). The control group consisted of 37 age- (64.78 ± 7.63) and sex- (M: F = 22:15) matched healthy controls (HC).

Statements on Ethics and the Declaration of Helsinki are given in the Supplement, as well as inclusion and exclusion criteria and clinical, behavioral, neuropsychological measures. MRI data acquisition, analysis and statistical analysis is also mentioned in the Supplement.

## Results

### Demographic, clinical and behavioral variables (Table 1)

**Table 1:** Represent the demographic, clinical and behavioral measures of 37 bvFTD and 37 age and-gender matched healthy controls

	Control (Mean ± SD)	Patient (Mean ± SD)	p-value
Age (years)	64.78 ± 7.63	63.81 ± 11.32	0.666
Sex (M: F)	22:15	21:16	0.93
Education (years)	15.32 ± 2.39	13.57 ± 3.12	0.008*
MMSE	29.30 ± 0.91	21.97 ± 7.27 (34)	< 0.001**
Disease Duration (years)	NA	1.51 ± 2.64	-
CDR-SOB	0.056 ± 0.21 (27)	10.13 ± 5.82 (34)	< 0.001*
NPI total	0.88 ± 1.9 (25)	11.06 ± 7.01 (34)	-<0.001*
^§^NPI Apathy	-	2.42 ± 0.68 (23)	-
^§^NPI_Disinhibition	-	1.95 ± 0.70 (21)	-
^§^GDS	0.97 ± 1.86 (31)	4.23 ± 3.85 (26)	< 0.001*
^§^HSCT (A) (nc)	14.79 ± 0.42 (19)	13.36 ± 3.23 (22)	0.053
^§^HSCT (B) (nc)	12.21 ± 2.27 (19)	6.43 ± 5.70 (21)	< 0.001**
^§^HSCT (B-A) (sec.)	41.79 ± 22.88 (19)	33.63 ± 45.16 (19)	0.108
^§^HSCT error (in category A)	0.21 ± 0.53 (19)	5.62 ± 5.44 (21)	< 0.001*
^§^HSCT error (in category B)	2.58 ± 2.22 (19)	1.90 ± 1.64 (21)	0.287
^§^DS Forward	7.94 ± 1.80 (35)	6.61 ± 1.94 (18)	0.021*
^§^DS Backward	6.0 ± 1.44 (35)	4.67 ± 2.30 (18)	0.031*

*Shows significant differences at p < 0.05, ** shows highly significant, ^§^represent the variables showing the maximum subjects in bracket with available scores for the corresponding test in each group (bvFTD patients as well as healthy controls). MMSE: mini mental screening examination; CDR-SOB: clinical dementia rating scale-sum of boxes; NPI: neuropsychiatric inventory; GDS: Geriatric depression scale; HSCT: Hayling sentence completion test; DS: Digit span; nc: number of correct sequences. M: male; F: female; nc: number of correct responses; sec.: seconds

Among the demographic variables, age and sex were comparable between groups while education was significantly higher in HC (t(72) = 2.72; p = 0.008). The bvFTD patients showed significantly lower scores for MMSE (t(69)=-5.83; p < 0.001) and high scores on the NPI apathy scale (2.42 ± 0.68). Response inhibition in section B of the Hayling Sentence Completion Test (HSCT) was impaired, as indicated by fewer correct (t(38)=-4.28; p < 0.001) and more automated responses (category A errors: (t(38) = 4.53; p < 0.001). Moreover, short-term memory span (digit span forward: t(51)=-2.42; p < 0.021) and working memory (digit span backward: t(51)=-2.29; p < 0.031) were reduced.

For the demographic data of sub cohort of 22 bvFTD with complementary resting fMRI data and 22 matched controls see the supplementary Table 1.

### Cortical thickness

**Fig. 1:**
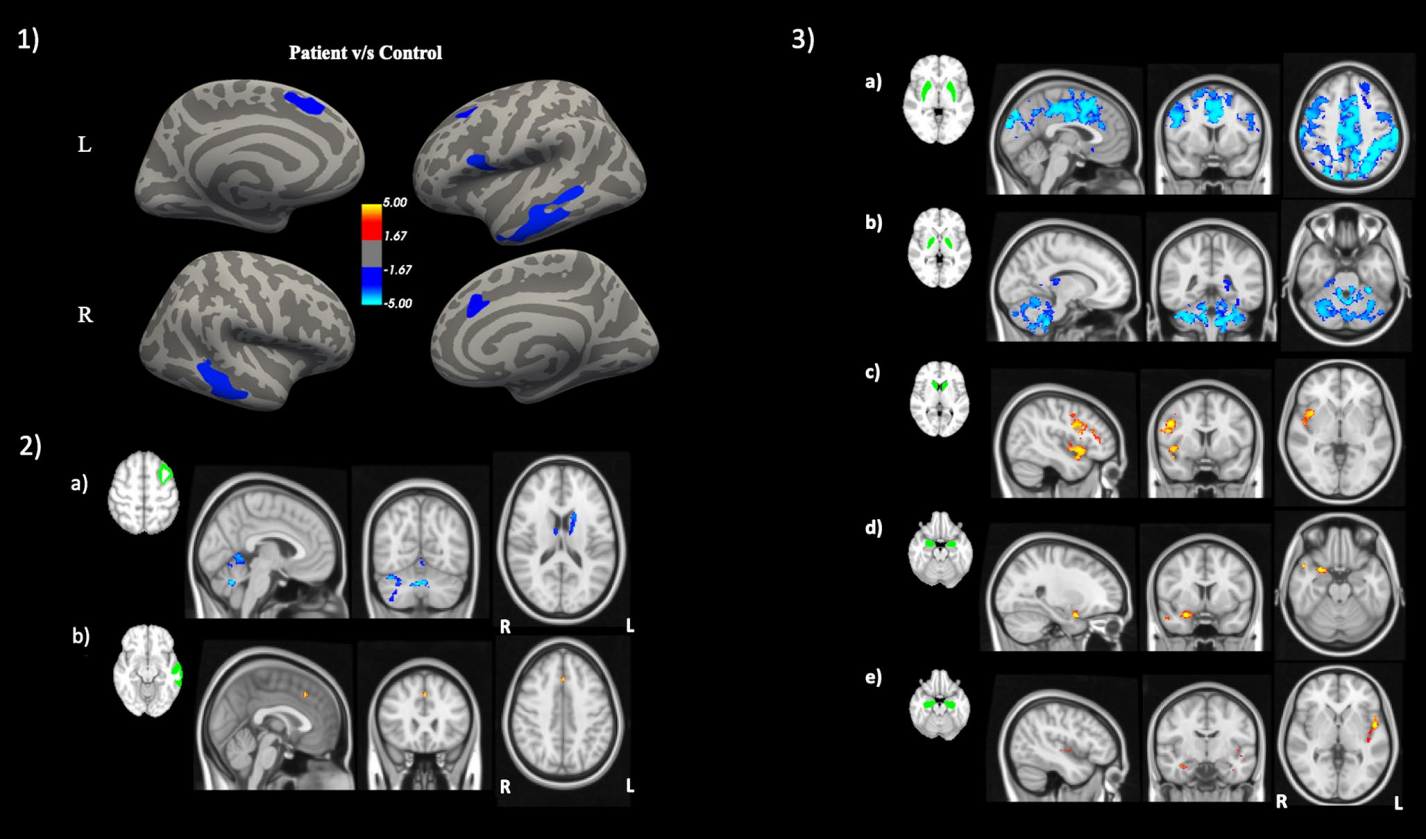
Display of structural changes in 37 bvFTD as compared to 37 healthy controls: 1.1) Decreased cortical thickness in 37 bvFTD (blue color) as well as Changes in FC when considered regions exhibiting cortical thinning and sub cortical volume loss as seeds in 22 bvFTD compared to 22 HC; 1.2a) Decreased FC with respect to left caudal middle frontal gyrus and; 1.2b) Increased FC with respect to left middle temporal gyrus; 1.3a) decreased FC with respect to putamen; 1.3b) decreased FC with respect to pallidum; and 1.3c) increased FC with respect to caudate nucleus; 1.3d) increased FC with respect to amygdala; and 1.3e) increased FC with respect to hippocampus. Here, decrease in FC is presented with blue color while increase with red-yellow. FC = functional connectivity; L = left; R = right

A significant bilateral cortical thinning was observed in bvFTD patient group in the left hemisphere, affecting caudal middle frontal gyrus (CMFG), middle temporal gyrus (MTG), pars opercularis and superior frontal gyrus (SFG). In the right hemisphere, only MTG and SFG showed significant cortical thinning (Fig. 1 part 1.1).

### Subcortical volume

A general linear model implementing age, sex, education, total intracranial volume and scanner site as nuisance variables showed significant bilateral volume losses in thalamus, caudate nucleus, putamen, pallidum, amygdala, hippocampus and nucleus accumbens in bvFTD (Supplementary Table 2).

### Cortical seeds-to-whole-brain FC

Among all cortically thinned regions only left CMFG and left MTG showed significant FC changes in bvFTD. Furthermore, left caudal middle frontal gyrus showed decreased FC with dorsal striatum, anterior thalamus and cerebellar regions (Fig. 1 part 1.2a). Conversely, left MTG showed increased FC with paracingulate gyrus (Fig. 1 part 1.2b).

### Subcortical seeds-to-whole-brain FC

For subcortical regions, decreased and increased FC was observed in bvFTD: Putamen showed decreased FC with cingulate, medial, lateral frontal and parietal cortex (Fig. 1 part 1.3a) and pallidum with medial and lateral cerebellar lobes (Fig. 1 part 1.3b). Conversely, caudate nucleus showed increased FC with right insula and inferior frontal gyrus (Fig. 1 part 1.3c). Amygdala and hippocampus showed increased FC with right parahippocampal gyrus, temporal pole and central opercular cortex (Fig. 1 parts 1.3d and 1.3e, respectively). No FC changes occurred in networks related to thalamus and nucleus accumbens seeds.

### Clinical correlations

Clinical correlations were assessed in 37 bvFTD patients, showing CDR-SOB to correlate positively with apathy subscores of the NPI-Q (r = 0.69, p < 0.001) and negatively with MMSE (r= -0.54, p = 0.011) in bvFTD.

### Clinico-radiological correlations

Clinico-radiological correlations between structural and clinical and neuropsychological scores were measured in 37 bvFTD. CDR-SOB scores correlated negatively with CTh measures in left MTG (r= -0.47; p = 0.004), left SFG (r= -0.52; p = 0.001), left CMFG (r= -0.47; p = 0.004), and right SFG (r= -0.62; p < 0.001). Correlations with NPI-Q sub-scores for apathy severity showed negative correlations with CTh in left pars opercularis (r=-0.54; p = 0.011), left CMFG (r=-0.54; p = 0.011), and right SFG (r=-0.57; p = 0.007). Moreover, in subcortical regions, only bilateral caudate nuclei showed trend negative correlations (r=-0.57, p = 0.007) with apathy severity of NPI-Q.

## Discussion

This multimodal MRI study investigated structural and functional changes and their association with neuropsychiatric symptoms, notably apathy, disinhibition and executive dysfunction in patients with bvFTD. Significant cortical thinning was found in frontal and temporal regions in bvFTD, along with subcortical volumetric reductions in all seven tested regions. Assessment of FC in seed regions obtained from structural analyses helped identifying functional changes associated with underlying structural loss: Decreased FC was found between left CMFG and anterior caudate nucleus and increased FC between left MTG and paracingulate gyrus. Similarly, subcortical seed regions also showed both decreased and increased FC in bvFTD. Putamen and pallidum showed decreased FC with fronto-parietal and cerebellar areas, respectively, while caudate nucleus, amygdala and hippocampus demonstrated increased FC with insula, inferior frontal and parahippocampus gyrus. Additionally, correlation analyses between clinical, behavioral and structural measures suggest that cortical thinning in frontal regions and volume loss in caudate nucleus relate with apathy severity in bvFTD.

Previous studies reported lateral and dorsal frontal areas to be predominantly linked with apathy in bvFTD (Moretti & Signori, 2016; Ducharme et al., 2018; Sheelakumari et al., 2020; Jenkins et al., 2022). We found cortical thinning in left CMFG, left pars opercularis, and right SFG to correlate with apathy severity. Middle frontal areas are involved in motivated behaviors (Kouneiher et al., 2009) whereas lateral frontal areas are more involved in cognitive control (Bahlmann et al., 2015). Thus, we speculate that the structural reductions cause amotivated behavior and reduced cognitive control (shown by higher scores for HSCT error of category A), possibly leading to apathetic behavior in bvFTD.

Affection of the basal ganglia also seems to play a distinctive role for apathy (Jenkins et al., 2022). Basal ganglia atrophy, especially of caudate nucleus, putamen and pallidum, has been reported in bvFTD (Bertoux et al., 2015; Macfarlane et al., 2015) and was reported to be relevant for apathy in bvFTD (Jenkins et al., 2022). Our observations of caudate atrophy in relation with increased apathy severity gives further support to the striatal role for apathy in bvFTD (Bertoux et al., 2015; Kumfor et al., 2018).

Beyond the independent role of the frontal cortex and the basal ganglia for apathy outlined above, fronto-striatal circuit impairment has been linked to apathy in elderly individuals (Hamada et al., 2021). The novelty of the current study is the demonstration of fronto-striatal circuit impairment, indexed as altered FC, in bvFTD. Notably, decreased FC between left CMFG and dorsal striatum was a principal finding, suggesting perturbation of fronto-striatal networks in bvFTD. Moreover, NPI-Q sub-scores for apathy severity were inversely correlated with cortical thickness in frontal and volume loss in striatal regions, strengthening the pathophysiological relevance of our multimodal approach in bvFTD.

In conclusion, the presented data indicate that structural alterations in frontal and striatal regions lead to disintegration of fronto-striatal networks in bvFTD and contribute to apathy severity, a core clinical symptom of the disease. Meanwhile, we acknowledge the small sample size and some missing behavioral measures. Future multimodal studies with bigger sample sizes and complete data sets warrant further validation of our observations.

### Electronic supplementary material

Below is the link to the electronic supplementary material.

Supplementary Material 1

## Data Availability

Due to limits on data distribution and usage imposed by patient agreement, the data used in the current investigation are not publicly accessible, although they may be obtained upon justifiable request from the DESCRIBE, DELCODE, and DANCER study consortia.

## Abbreviations

bvFTD: Behavioral variant frontotemporal dementia
CDR-SOB: Clinical Dementia Rating Scale - Sum Of Boxes
FTD: Frontotemporal dementia
FC: Functional connectivity
HSCT: Hayling Sentence Completion Test
MRI: Magnetic resonance imaging
MMSE: Mini Mental Screening Examination
NPI-Q: Neuropsychiatric Inventory Questionnaire
nc: Number count
rsfMRI: Resting-state functional magnetic resonance imaging
