# Supplementary material for: Fronto-striatal alterations correlate with apathy severity in behavioral variant frontotemporal dementia
Source: Brain Imaging Behav. 2023 Oct 19;18(1):66–72. doi: 10.1007/s11682-023-00812-3 (PMC10844138; doi:10.1007/s11682-023-00812-3)
Supplement: Supplementary file 1 — Supplementary Material 1 [file 11682_2023_812_MOESM1_ESM.docx]

Methods:

Recruitment: DESCRIBE (DZNE Clinical Register Study of Neurodegenerative Disorders): All behavioral variant Frontotemporal Dementia (bvFTD) patients were assessed according to routine medical care at the respective centers (including medical history, psychiatric and neurological examination, blood laboratory work-up, and MRI) before entering DESCRIBE. For inclusion into the DESCRIBE-FTD subcohort, participants had to be >18 years, fulfill the established diagnostic criteria for a clinical FTD variant (or present as a symptomatic carrier of FTD-related genetic mutations), and provide written informed consent (by the patient, or a legal representative). Exclusion criteria were instable or severe medical conditions, previous or current psychological, psychiatric and physical illnesses which precluded study participation, as well as a clinical diagnosis of corticobasal degeneration or progressive supranuclear palsy (and for the present study: MR-related contraindications).

The clinical assessments included a structured medical history, current medication, structured family history, standardized physical examination, including sensory testing, the Mini-Mental State Examination (MMSE) (Folstein et al., 1975), Clinical Dementia Rating (CDR) sum of boxes (CDR-SOB) (Borroni et al., 2010), the 15-item short form of the Geriatric Depression Scale (GDS) (Sheikh & Yesavage, 1986). Informants/caregivers were interviewed with the Neuropsychiatric Inventory (NPI-Q) (Cummings, 1997) to assess the neuropsychiatric symptoms.

Degeneration Controls and Relatives (DANCER): This ongoing study of the German Center of Neurodegenerative Diseases (DZNE) recruits healthy controls (HC) among family members and friends of patients with neurodegenerative disorders, as well as individuals without known neurodegenerative disease who respond to public calls and advertisements. Inclusion criteria were age ≥ 18 years, fluent German language skills, and written informed consent. We excluded individuals who reported instable or severe medical conditions, previous or current psychological, psychiatric and physical illnesses which precluded study participation, showed cognitive problems in clinical testing (MMSE<26) and, for the present analysis, MRI contradictions. Among N=21 DANCER participants included ongoing study recruitment, projects, N=10 were patient relatives and N=10 advertised (N=1 with missing information). We cannot exclude the risk that some of the former participants were asymptomatic FTD-mutation carriers, since no genetic characterization was available for this sample. Meanwhile, this means that observed group differences between bvFTD patients and controls should actually provide a conservative estimate of the actual differences.

Since the DANCER participants are recruited as a comparison group for DESCRIBE patients, the clinical and neuropsychological assessment protocols are largely overlapping.

To match age differences from the DESCRIBE bvFTD cohort, another N=16 HC participants from the DELCODE study, which focuses on the preclinical stage of Alzheimer’s disease (Jessen et al., 2018): These participants were recruited via advertisement, had to be >=60 years, show fluent German language skills, negate cognitive problems, present unimpaired neuropsychological test performance), and should not report a first-degree relative with dementia. Similar to DESCRIBE and DANCER, exclusion criteria included instable or severe medical conditions, previous or current psychological, psychiatric and physical illnesses which precluded study participation, and neurodegenerative diseases other than Alzheimer’s Disease. Detailed inclusion and exclusion criteria are provided elsewhere (Jessen et al., 2018).

Again, genetic characterization of FTD-related mutations was not available for these participants. Moreover, the clinical and neuropsychological assessment batteries for DELCODE were overlapping, but not identical with DESCRIBE and DANCER, meaning that some behavioural measures were not available for these control participants.

Individuals were informed about the study protocol and signed the consent. The study was approved by the local ethics committees of the participating sites under coordination of the University Hospital Bonn (Medical Faculty of Rheinische Friedrich-Wilhelms-Universität Bonn: Lfd. Nr. 311/14 (DESCRIBE), 117/13 (DELCODE), 312/14 (DANCER)), and in accordance with national legislation and the Declaration of Helsinki.

Neuropsychological measures

A German adaptation of the Hayling Sentence Completion Test (*Burgess, P. & Shallice, T. (1997)* was used to measure cognitive inhibition (Montembeault et al., 2020). It consists of two sentence completion tasks: 1) using appropriate word (automatic condition, section A), and 2) using completely unconnected word (inhibition condition, section B). In each condition, participants listen to 15 sentences, in which the last word is missing. In the first condition (section A) individuals had to complete sentences as fast as possible with a semantically correct word clearly suggested by the context; in the second condition (section B) individuals had to complete the sentences with a non-sense word, which requires them to suppress their automatic, semantically correct response. There are different outcomes of this test: latency, number of correct responses in each trial as well as the Hayling Time B-A (total time to complete sentences in section A subtracted from the total time to complete sentences in section B) and the Hayling error scores (reflecting category A and category B errors in section B). For section B, responses that might have reasonably finished the statement were classified as category A errors. Responses that were indirectly related to the statement but did not directly complete it fell under category B errors. Correct replies included sentence completions that had nothing to do with the original text.

The German version of the digit span (DS) subtest in the Wechsler Memory Scale-Revised (Härting & Wechsler, 2000) was performed. This test includes two scales DS forward as a measure of short-term memory span while DS backwards served as a measure of working memory, another prototypical aspect of executive function (Miyake et al., 2000). In each condition, maximum number of correct responses was twelve.

MRI acquisition

Data was acquired using 3 Tesla Siemens scanners at multiple study sites (two TrioTim, three Verio, two Skyra, and one Prisma). Most of the scanners used 32-channel head coil while only two used 20 channels, with harmonized sequence parameters. We acquired a T1- weighted magnetization-prepared rapid gradient echo with following sequence parameters: TR: 2500ms, TE: 4.37ms, TI: 1100ms, flip angle: 7°, GRAPPA = 2, FOV: 256 x 256 mm2, slice thickness: 1mm, 192 sagittal sections, no gap. Moreover, rsfMRI data were acquired with axial orientation using an echo-planar imaging with following sequence parameters: TR/TE = 2580/ 30 ms, FOV = 224 × 224 mm2, flip angle: 80°, matrix size= 64 × 64, number of slices = 47, slice thickness = 3.5 mm, total of 180 volumes in 8 min duration. During rsfMRI, subjects were instructed to keep their eyes closed, not to fall asleep, hold still, and avoid focusing on specific thoughts.

Data analysis

Structural MRI

T1-weighted images were analyzed using the surface-based stream in FreeSurfer (https://surfer.nmr.mgh.harvard.edu/fswiki) to obtain CTh and subcortical volumes. It included motion correction, Talairach transformation, removal of nonbrain tissues, and automated segmentation of data into gray and white matter (Dale et al., 1999). Subsequently, cortical inflation, registration to a spherical atlas, and parcellation of cerebral cortex into gyral and sulcal units were performed (Desikan et al., 2006). Labeling of cortical regions was performed on the basis of the Desikan/Killiany atlas (Destrieux et al., 2010). CTh was calculated by measuring the closest distance from pial to white matter boundary at each vertex (Fischl & Dale, 2000).

Additionally, automated volumetric processing based on a probabilistic atlas in FreeSurfer generated subcortical labels (Fischl et al., 2002). From these labels, volumetric measures of seven subcortical gray matter structures (thalamus, caudate nucleus, nucleus accumbens, pallidum, putamen, hippocampus, and amygdala) were extracted in each hemisphere.

Resting state fMRI:

A subgroup of 22 patients had complementary rsfMRI data, hence, 22 matched healthy controls were included in the FC analysis. Preprocessing of rsfMRI data was performed using the FSL toolbox pipeline (https://fsl.fmrib.ox.ac.uk/fsl/fslwiki/FEAT). A seed-based whole brain FC analysis was performed using filtered clean rsfMRI data in standard space using FSL toolbox.It included individual motion and distortion correction using Mcflirt and fieldmaps, respectively. Individual functional data were non-linearly registered to corresponding T1-weighted images. Data were spatially smoothed (5mm) and high-pass filtered (>0.01Hz). Independent components were extracted at the individual level for further denoising. Denoising was performed using the ICA-FIX method in order to reduce multisite differences (Feis et al., 2015): first, we created signal and noise labels for individual ICA maps in 10 subjects. These labels were than used to train model and further applied to each individual ICA maps to create signal and noise labels. Finally, we regressed out the noise components and the motion parameters using regression filter available in FSL.

A seed-based whole-brain FC analysis was performed using filtered clean rsfMRI data in standard space using FSL toolbox. Seeds were created from the brain areas showed significant differences in CTh and subcortical volume between bvFTD and healthy controls. Dual regression was performed over the whole-brain using each seed mask in order to obtain respective FC maps.

Statistical analysis:

Demographic, clinical, behavioral and subcortical volume measures were analyzed using SPSS Version 25.0 (Armonk, NY: IBM Corp.). Variables showing normal distributions were analyzed using a two-sample t-test while others with Mann-Whitney U test for comparison between groups. Categorical variables (i.e., sex) were compared using a chi square test.

CTh maps were smoothed (10mm) and inserted into a general linear model to compare between groups including age, sex, education, total intracranial volume and acquisition sites as nuisance variables. CTh changes were obtained at p<0.05, corrected for cluster-level familywise error using Monte-Carlo simulation.

Subcortical volumes from seven regions were also compared between groups using general linear model including age, sex, education, total intracranial volume, and scanner site as covariates of no interest. Significant results were reported at p<0.05, corrected for multiple comparisons using a Bonferroni approach.

Seed-to-whole brain FC maps were inserted into a general linear model included age, sex, education, and scanner site as covariates of no interests to assess group differences. A randomise approach reported significant clusters at p<0.05, after correcting for multiple comparisons using threshold-free cluster enhancement.

An exploratory Pearson correlation was performed between clinical, behavioral and Cortical thickness and subcortical volume measures obtained from freesurfer analyisis using SPSS. Significant correlations were reported at p<0.05 for cortical regions and at p<0.005 for subcortical regions (corrected for multiple comparisons using number of subcortical regions (0.05/10 =0.005)). We also reported non-significant correlations (0.05>p>0.005) as trend. We did not perform correlation analysis between clinical, behavioral and FC changes, as there was a high proportion of missing values of clinical and behavioral measures leading to a too small sample size.

Results

**Supplementary Table1.** Represent the demographic, clinical and behavioral measures of 22 bvFTD patients and matched controls for whom complementary resting fMRI data was available in the original cohort mentioned in Table1. It also shows significant differences after implementing the Mann-Whitney U test.

|  | CONTROL  (Mean±SD) | bvFTD  (Mean±SD) | pvalue |
| --- | --- | --- | --- |
| Age | 63.41±11.33 | 62.45±11.0 | 0.197 |
| Sex (M: F) | 13:9 | 14:8 | 0.757 |
| Education | 15.14±2.43 | 13.54±3.16 | 0.085 |
| MMSE | 29.23±1.23 | 22.81±6.44 (21) | <0.001** |
| ^§^CDR-SOB | 0.12±0.28 (17) | 6.29±4.18 (21) | <0.001** |
| ^§^NPI total | 1.36±2.47 (14) | 9.71±6.89 (21) | <0.001* |
| ^§^NPI_Apathy | - | 2.08±0.64 (13) |  |
| ^§^NPI_Disinhibition | - | 2.27±0.65 (11) |  |
| ^§^GDS | 1.12±1.83 (17) | 5.0±4.44 (15) | 0.005 |
| ^§^HSCT (A) ^nc^ | 14.80±0.42 (10) | 13.29±3.93 (14) | 0.110 |
| ^§^HSCT (B) ^nc^ | 14.20±1.32 (10) | 6.93±5.89 (14) | <0.001** |
| ^§^HSCT (B-A) (sec.) | 28.20±21.22 (10) | 45.71±45.32 (14) | 0.617 |
| ^§^HSCT error (in category A) | 0.10±0.32 (10) | 4.79±5.38 (14) | 0.007 |
| ^§^HSCT error (in category B) | 0.70±1.34 (10) | 1.857±1.834 (14) | 0.046 |
| ^§^DS Forward | 8.48±2.23 (21) | 6.82±2.09 (11) | 0.014* |
| ^§^DS Backward | 6.29±1.74 (21) | 4.91±2.66 (11) | 0.018* |

*Shows significant differences at p<0.05, ** shows highly significant, ^§^represent the variables showing the maximum subjects in bracket with available scores for the corresponding test in each group (bvFTD patients as well as healthy controls). MMSE: mini mental screening examination; CDR-SOB: clinical dementia rating scale-sum of boxes; NPI: neuropsychiatric inventory; GDS: Geriatric depression scale; HSCT: Hayling sentence completion test; DS: digit span; nc: number of correct sequences. M: Male; F: female; nc: number of correct responses;sec.: seconds.

**Supplementary Table 2**. Comparisons obtained in subcortical volumes, after implementing a general linear model with age, sex, education, total-intracranial volume, and scanner site as a covariate of no interest between37 bvFTD patients and 37 matched healthy controls.

| Subcortical region | Controls  (Mean±SD) cm^3^ | bvFTD  (Mean±SD) cm^3^ | p-value | Cohen’s d Effect Size |
| --- | --- | --- | --- | --- |
| Left Thalamus | 6.66±0.75 | 6.01±1.14 | 0.001 | 0.674 |
| Right Thalamus | 6.73±0.62 | 6.14±1.08 | <0.001 | 0.67 |
| Left Caudate Nucleus | 3.36±0.51 | 2.81±0.78 | 0.001 | 0.835 |
| Right Caudate Nucleus | 3.56±0.54 | 2.94±0.85 | 0.001 | 0.871 |
| Left Putamen | 4.35±0.52 | 3.67±0.75 | <0.001 | 1.054 |
| Right Putamen | 4.45±0.49 | 3.76±0.79 | <0.001 | 1.05 |
| Left Pallidum | 1.98±0.26 | 1.72±0.42 | <0.001 | 0.744 |
| Right Pallidum | 1.79±0.24 | 1.56±0.35 | <0.001 | 0.766 |
| Left Hippocampus | 3.80±0.36 | 3.22±0.61 | <0.001 | 1.158 |
| Right Hippocampus | 3.98±0.43 | 3.29±0.67 | <0.001 | 1.226 |
| Left Amygdala | 1.47±0.21 | 1.15±0.32 | <0.001 | 1.182 |
| Right Amygdala | 1.58±0.25 | 1.30±0.31 | <0.001 | 0.994 |
| Left Nucleus Accumbens | 0.36±0.09 | 0.24±0.11 | <0.001 | 1.194 |
| Right Nucleus Accumbens | 0.47±0.10 | 0.33±0.13 | <0.001 | 1.207 |

The significant differences corrected for Bonferroni corrections (<0.05/14=0.004).

References:

Borroni, B., Agosti, C., Premi, E., Cerini, C., Cosseddu, M., Paghera, B., Bellelli, G., & Padovani, A. (2010). The FTLD-modified Clinical Dementia Rating scale is a reliable tool for defining disease severity in frontotemporal lobar degeneration: Evidence from a brain SPECT study. *European Journal of Neurology*, *17*(5), 703–707. https://doi.org/10.1111/j.1468-1331.2009.02911.x

*Burgess, P. & Shallice, T. (1997) The Hayling and Brixton Tests. Test manual. Bury St Edmunds, UK: Thames Valley Test Company.* (n.d.).

Cummings, J. L. (1997). The Neuropsychiatric Inventory: Assessing psychopathology in dementia patients. *Neurology*, *48*(5 Suppl 6), S10-16. https://doi.org/10.1212/wnl.48.5_suppl_6.10s

Dale, A. M., Fischl, B., & Sereno, M. I. (1999). Cortical surface-based analysis. I. Segmentation and surface reconstruction. *NeuroImage*, *9*(2), 179–194. https://doi.org/10.1006/nimg.1998.0395

Desikan, R. S., Ségonne, F., Fischl, B., Quinn, B. T., Dickerson, B. C., Blacker, D., Buckner, R. L., Dale, A. M., Maguire, R. P., Hyman, B. T., Albert, M. S., & Killiany, R. J. (2006). An automated labeling system for subdividing the human cerebral cortex on MRI scans into gyral based regions of interest. *NeuroImage*, *31*(3), 968–980. https://doi.org/10.1016/j.neuroimage.2006.01.021

Destrieux, C., Fischl, B., Dale, A., & Halgren, E. (2010). Automatic parcellation of human cortical gyri and sulci using standard anatomical nomenclature. *NeuroImage*, *53*(1), 1–15. https://doi.org/10.1016/j.neuroimage.2010.06.010

Feis, R. A., Smith, S. M., Filippini, N., Douaud, G., Dopper, E. G. P., Heise, V., Trachtenberg, A. J., van Swieten, J. C., van Buchem, M. A., Rombouts, S. A. R. B., & Mackay, C. E. (2015). ICA-based artifact removal diminishes scan site differences in multi-center resting-state fMRI. *Frontiers in Neuroscience*, *9*, 395. https://doi.org/10.3389/fnins.2015.00395

Fischl, B., & Dale, A. M. (2000). Measuring the thickness of the human cerebral cortex from magnetic resonance images. *Proceedings of the National Academy of Sciences of the United States of America*, *97*(20), 11050–11055. https://doi.org/10.1073/pnas.200033797

Fischl, B., Salat, D. H., Busa, E., Albert, M., Dieterich, M., Haselgrove, C., van der Kouwe, A., Killiany, R., Kennedy, D., Klaveness, S., Montillo, A., Makris, N., Rosen, B., & Dale, A. M. (2002). Whole brain segmentation: Automated labeling of neuroanatomical structures in the human brain. *Neuron*, *33*(3), 341–355.

Folstein, M. F., Folstein, S. E., & McHugh, P. R. (1975). “Mini-mental state”. A practical method for grading the cognitive state of patients for the clinician. *Journal of Psychiatric Research*, *12*(3), 189–198. https://doi.org/10.1016/0022-3956(75)90026-6

Härting, C., & Wechsler, D. (2000). *Wechsler-Gedächtnistest—Revidierte Fassung WMS-R ; Manual ; deutsche Adaptation der revidierten Fassung der Wechsler Memory scale*.

Jessen, F., Spottke, A., Boecker, H., Brosseron, F., Buerger, K., Catak, C., Fliessbach, K., Franke, C., Fuentes, M., Heneka, M. T., Janowitz, D., Kilimann, I., Laske, C., Menne, F., Nestor, P., Peters, O., Priller, J., Pross, V., Ramirez, A., … Düzel, E. (2018). Design and first baseline data of the DZNE multicenter observational study on predementia Alzheimer’s disease (DELCODE). *Alzheimer’s Research & Therapy*, *10*(1), 15. https://doi.org/10.1186/s13195-017-0314-2

Miyake, A., Friedman, N. P., Emerson, M. J., Witzki, A. H., Howerter, A., & Wager, T. D. (2000). The unity and diversity of executive functions and their contributions to complex “Frontal Lobe” tasks: A latent variable analysis. *Cognitive Psychology*, *41*(1), 49–100. https://doi.org/10.1006/cogp.1999.0734

Montembeault, M., Sayah, S., Rinaldi, D., Le Toullec, B., Bertrand, A., Funkiewiez, A., Saracino, D., Camuzat, A., Couratier, P., Chouly, M., Hannequin, D., Aubier-Girard, C., Pasquier, F., Delbeuck, X., Colliot, O., Batrancourt, B., Azuar, C., Lévy, R., Dubois, B., … PrevDemAls study group. (2020). Cognitive inhibition impairments in presymptomatic C9orf72 carriers. *Journal of Neurology, Neurosurgery, and Psychiatry*, *91*(4), 366–372. https://doi.org/10.1136/jnnp-2019-322242

Sheikh, J. I., & Yesavage, J. A. (1986). Geriatric Depression Scale (GDS): Recent evidence and development of a shorter version. *Clinical Gerontologist: The Journal of Aging and Mental Health*, *5*(1–2), 165–173. https://doi.org/10.1300/J018v05n01_09
